# Short-Term Clinical Outcomes of Patients with Diabetic Macular Edema Following a Therapy Switch to Faricimab

**DOI:** 10.3390/jcm13154508

**Published:** 2024-08-01

**Authors:** Peter Wolfrum, Elsa Wilma Böhm, Katrin Lorenz, Bernhard Stoffelns, Norbert Pfeiffer, Christina A. Korb

**Affiliations:** Department of Ophthalmology, University Medical Center, Johannes Gutenberg-University Mainz, Langenbeckstr. 1, 55131 Mainz, Germany

**Keywords:** diabetic retinopathy, diabetic macular edema, diabetes, anti-VEGF, retina, DME, faricimab

## Abstract

**Background:** With this study, we investigate the short-term clinical outcomes of patients affected by diabetic macular edema (DME) after switching to intravitreal Faricimab (IVF) in a real-world setting. **Methods:** We conducted a retrospective chart review on all patients treated for DME with IVF who showed insufficient responses to prior anti-VEGF therapy. Data collected included baseline patient demographics, medical history, best-corrected visual acuity (BCVA), central retinal thickness (CRT) and central retinal volume (CRV). We analyzed functional and structural measures before and after IVF, compared baseline demographics and treatment factors between Faricimab-responders and reduced-responders and assessed influencing factors of the follow-up BCVA and CRT. **Results:** This study included 25 eyes from 16 patients. After switching to IVF, the mean BCVA showed no significant improvement, changing from 59.4 ± 13.4 Early Treatment of Diabetic Retinopathy Study (ETDRS) letters at baseline to 61.4 ± 12.8 ETDRS letters at follow-up (*p* = 0.26). CRT significantly reduced from 414.4 ± 126.3 µm to 353.3 ± 131.1 µm (*p* < 0.011), and the 3 mm CRV significantly decreased from 2.8 ± 0.5 mm^3^ to 2.6 ± 0.6 mm^3^ (*p* < 0.012). Seven patients met the responder criteria, exhibiting an improvement of at least 5 ETDRS letters and a simultaneous CRT reduction of at least 30 µm. Further analysis showed that higher BCVA at baseline (*p* < 0.001) was associated with better BCVA following IVF, while higher baseline CRT (*p* < 0.003), a higher number of prior anti-VEGF agents (*p* < 0.034) and prior corticosteroid injections (*p* < 0.019) were associated with greater CRT at follow-up. **Conclusions:** Following the initial IVF injection series, we observed a clear improvement of anatomical measures. No functional improvement was observed, although visual acuity remained stable. Higher baseline BCVA was associated with better post-IVF BCVA, while higher baseline CRT, a greater number of prior anti-VEGF agents and prior corticosteroid injections were linked to higher CRT post-IVF.

## 1. Introduction

Diabetic retinopathy (DR) constitutes the leading cause of vision loss in working-age adults, as well as the third overall cause of blindness in industrialized countries. A worldwide occurrence of around 93 million people is expected to be affected by DR, whereas a worrying rising prevalence has been especially reported in developing countries in the past decade [1].

DR is a secondary microvascular disease, following diabetes, that occurs due to long-term elevated blood glucose levels, leading to the activation of several cellular pathways, increased formation of advanced glycation end products, increased oxidative stress as well as endothelial dysfunction [2]. As a result, damage to the retinal vasculature occurs, leading to microaneurysms, intraretinal bleedings and swellings. Advanced stages can also lead to neovascular complications and abnormal blood vessel growth, vitreous hemorrhage as well as proliferative vitreous complications and tractional retinal detachments. Good blood glucose control and regular eye examinations are crucial for the early detection and management of this disease [3]. Concomitant cardiovascular risk factors, such as dyslipidemia or hypertension, further aggravate microvascular complications and disease progression [4,5]. However, it has been shown that prior intensive glycemic control reduces DR progression [6]. Moreover, the intense treatment of cardiovascular risk factors, such as dyslipidemia, has also been shown to have beneficial effects on DR progression [7,8].

Diabetic macular edema (DME) is a serious complication of DR also leading to significant visual impairment if left untreated. Similar to the treatment of neovascular age-related macular degeneration (nAMD), the first-line treatment for DME usually involves intravitreal injections of anti-vascular-endothelial-growth-factor (VEGF) drugs such as Bevacizumab, Aflibercept, Ranibizumab or Brolucizumab. Sometimes, individuals suffering from DME do not show sufficient responsiveness to particular anti-VEGF agents. This situation necessitates considering either switching to a different anti-VEGF agent or the injection of an intravitreal corticosteroid, given the restricted availability of approved anti-VEGF drugs for medical intervention.

In January 2022, Faricimab (Roche, Basel, Switzerland), a novel bispecific antibody-based agent for the treatment of nAMD as well as DME, was approved by the FDA. Faricimab represents a monoclonal antibody simultaneously addressing the vascular endothelial growth factor A (VEGF-A) as well as angiopoietin 2 (Ang-2). Ang-2 is a key regulator during angiogenesis by the negative regulation of angiopoietin 1/tyrosine kinase with immunoglobulin (Ig) and epidermal growth factor (Tie) 2 signaling. It has been shown to be upregulated under pathological conditions, and by the inhibition of the endothelial-specific Tie 2 receptor, it potentiates the action of VEGF. By the promotion of detachment of pericytes, decreased integrity of endothelial cell junctions and recruitment of inflammatory cells, vascular instability increases and aggravates retinal vascular disease [9]. Direct aspects of Ang-2 on vascular instability have also been reported previously. By direct effects on integrin signaling, endothelial destabilization and angiogenesis are promoted [10,11]. Several preclinical studies analyzed the synergistic effect of Ang-2 and VEGF. The dual inhibition was found to more effectively reduce photoreceptor apoptosis, retinal inflammation and choroidal neovascularization lesion leakage in animal models, compared to the blockade of VEGF-A or Ang-2 alone [9,12]. In patients with proliferative diabetic retinopathy undergoing pars plana vitrectomy, significantly upregulated levels of VEGF-A and Ang-2 were detected [13,14].

The dual mechanism of action of Faricimab showed promising results in the YOSEMITE and RHINE trial [15]. Through improvements of the baseline visual acuity, the retinal structure and prolonged intertreatment intervals could have been preserved up to 2 years [15].

In comparison to controlled clinical trials, it has been shown many times that in the real-world setting, the clinical outcomes of anti-VEGF therapy have been inferior, which marks the importance of real-world data [16,17]. Suboptimal real-world outcomes can result, e.g., from under-treatment due to the burden of frequent injections and monitoring visits, as well as inconsistent adherence to long-term therapy [17,18]. Currently, only few real-world studies about the treatment outcomes, especially following a prior anti-VEGF therapy, of intravitreal Faricimab (IVF) in patients affected by DME have been published [19,20,21,22]. 

With this study, we therefore want to further explore the short-term clinical outcomes, as well as potential influencing factors, of an anti-VEGF therapy switch to Faricimab, following a prior anti-VEGF therapy in patients affected by DME in a real-world setting.

## 2. Methods

### 2.1. Ethical Approval and Consent

Due to the retrospective approach of this analysis, no ethical approval was required, and no informed consent was obtained from the patients, since no patient was prospectively involved in this study. Only the routine health data of patients treated in the Department of Ophthalmology, University Medical Center Mainz were investigated by retrospective chart review. No so-called third persons were allowed to have a direct inspection of the original health data of individual patients, and the publication was performed with anonymized data only. This type of investigation is regulated by the so-called “Landeskrankenhausgesetz, §§36-37”. This study was performed in accordance with the Declaration of Helsinki.

### 2.2. Patient Selection

For this analysis, all patients treated for DME in the Department of Ophthalmology, University Medical Center Mainz, Germany, who had received a prior anti-VEGF therapy and were switched to IVF between October 2022 and January 2024, were considered for inclusion (33 patients). Patients were treated under a pro re nata therapy scheme and had received a switch to IVF after previously showing an insufficient response to at least one prior anti-VEGF agent. Insufficient response was thereby determined by the physician in charge of the injection consultation but insufficient reduction in the central retinal thickness (CRT) as well as visual acuity deterioration under prior anti-VEGF-therapy was mainly considered. Exclusion criteria were treatment-naïve patients, missing relevant data, treatment discontinuities, as well as the presence of other retinal comorbidities such as nAMD, condition after vascular occlusion or chorioretinopathia centralis serosa.

### 2.3. Data Collection

The data collection was performed by two ophthalmologists (P.W. and C.K.). The baseline examination was defined as the day of the first injection of IVF, and the follow-up examination, 4 weeks after the last injection of the IVF series. Epidemiologic data from each patient included age, sex, laterality, type of diabetes, current HbA1c, status of diabetic retinopathy, number of prior intravitreal anti-VEGF-, corticosteroid- and triamcinolone-injections, time span since first injection, ocular and systemic comorbidities, history of prior eye surgeries and laser interventions, number of Faricimab injections as well as adverse events following IVF.

We collected the decimal best-corrected-visual-acuity (BCVA) at baseline, before every further IVF injection as well as during the follow-up visits. For analysis, the BCVA was converted to the Early Treatment Diabetic Retinopathy Study (ETDRS) letter score according to Beck et al. [23,24].

We analyzed SD-OCT images (Spectralis^TM^, Heidelberg Engineering, Heidelberg, Germany) at the defined baseline as well as follow-up exams. The CRT and the 3 mm central retinal volume (CRV) were measured with the software-included measurement tools of Heyex 2 (Heidelberg Engineering, Heidelberg, Germany).

### 2.4. Injection Procedure

The IVF injections were performed in an outpatient setting. Ocular surface anesthesia was applied (1% tetracaine eye drops), following the insertion of an eyelid speculum as well as the disinfection of the ocular surface and eyelid area (5% povidone–iodine disinfection). Using a 30-G needle, 6 mg/0.05 mL of Faricimab (Roche, Basel, Switzerland) was injected intravitreally via the pars-plana.

### 2.5. Outcomes

The primary outcome measures of this study were structural and functional outcome changes at the follow-up visit, 4 weeks after the last IVF injection compared to the baseline visit. The CRT and CRV were representative for structural measures, and the visual acuity was applied as functional measure. For the secondary outcome measures, we performed a subgroup analysis to further investigate differences in patient baseline characteristics between Faricimab responders and reduced-responders. For that comparison, the threshold of the responder group was set as a minimum reduction of the CRT of at least 30 µm as well as a simultaneous gain of at least 5 ETDRS letters at the follow-up compared to the baseline exam, similar to a previous treatment responder analysis by Ziemssen et al. [25]. The tertiary outcome measures included an exploratory correlation analysis to further investigate the influence of age, status of diabetic retinopathy, baseline CRT, baseline ETDRS letter score, years since first anti-VEGF injection, number of prior anti-VEGF injections and agents as well as number of prior corticosteroid injections on the follow-up CRT as well as follow-up ETDRS letter score.

### 2.6. Statistical Analysis

We performed the statistical analysis using SPSS Version 27 (IBM, New York, NY, USA). Wilcoxon signed-rank tests were used to compare the mean values of CRT, CRV and ETDRS letter score between the baseline and follow-up exam. For the subgroup analysis, the individuals were classified after the previously mentioned criteria. Mean clinical and demographical patient characteristics were compared between the groups using chi-squared tests for nominal measurements. For metric data, depending on the normality of distribution (according to the Kolmogorov–Smirnov and Shapiro–Wilk test), independent *t*-tests or Wilcoxon-Mann–Whitney-tests were performed. For the employed exploratory analysis, a Pearson or Spearman correlation was performed depending on the measurement between the previously mentioned baseline characteristics and follow-up CRT as well as the follow-up ETDRS letter score to investigate possible influencing factors. Statistical significance was defined by a *p*-value of <0.05.

## 3. Results

A total of 25 eyes of 16 patients met the inclusion criteria. The patients, 3 females and 13 males, had a mean age of 65 years, an average baseline HbA1c of 7.5% and received their first anti-VEGF injection approximately 4.6 years before their first IVF injection. A mean of 24.6 prior intravitreal anti-VEGF injections were applied before the therapy switch, with an average of 1.8 prior anti-VEGF agents. While no eyes with a mild non-proliferative diabetic retinopathy (NPDR) were included, 5 eyes with a moderate NPDR, 15 eyes with a severe NPDR as well as 5 eyes with a proliferative diabetic retinopathy (PDR) were included. On average, 3 IVF injections were applied in the initial series, and the follow-up exam was performed, on average, 3.26 months after the first IVF injection. A summary of the complete baseline characteristics of the entire study population is listed in Table 1.

**Figure 1:**
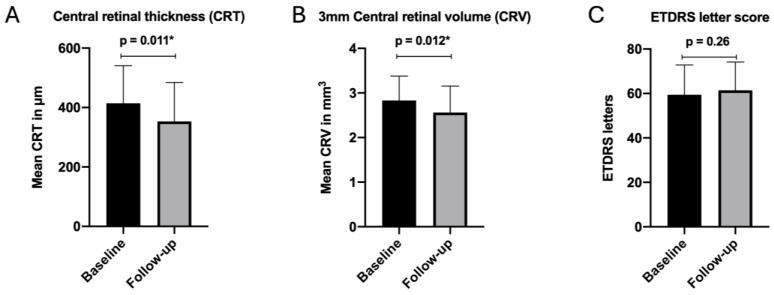
Primary outcome measures showing a significant reduction of the follow-up CRT (**A**) and CRV (**B**), following IVF. No significant change of the ETDRS letter score (**C**) was observed following IVF. Complete data included in Table S1 (Supplementary). Abbreviations: ETDRS, Early Treatment of Diabetic Retinopathy Study; IVF, intravitreal Faricimab. * Statistically significant.

The ETDRS letter score changed from 59.4 ± 13.4 letters at baseline to 61.4 ± 12.8 letters at follow-up, showing no significant improvements (*p* = 0.26). CRT experienced a notable reduction from 414.4 ± 126.3 µm at baseline to 353.3 ± 131.1 µm during the follow-up exam, which proved statistical significance (*p* < 0.011). The 3 mm CRV also showed a significant reduction from 2.8 ± 0.5 mm^3^ at baseline to 2.6 ± 0.6 mm^3^ at follow-up (*p* < 0.012). A graphical summary of the primary outcome measures is shown in Figure 1.

For the subgroup analysis, 7 of the total of 25 eyes met the responder criteria, while 18 did not exhibit a distinct simultaneous improvement in the previously appointed functional and structural aspects. 

The responder group had an average baseline ETDRS score of 52.9 ± 18.5 letters and experienced a mean gain of 10 ± 7.6 letters at the follow-up. Conversely, the reduced-responder group displayed a baseline ETDRS score of 61.9 ± 10.5 letters and experienced a mean loss of −1.1 ± 4.7 letters at follow-up. The average CRT in the responder group measured 464.6 ± 113.1 µm, with a mean reduction of −194.4 ± 99.3 µm; meanwhile, the reduced-responder group displayed a baseline CRT of 394.4 ± 128.7 µm, with a reduction of −9.3 ± 49.4 µm. In the responder group, the 3 mm CRV measured 3.1 ± 0.5 mm^3^, demonstrating a decline of −0.86 ± 0.4 mm^3^, while the reduced-responder group had a mean CRV of 2.7 ± 0.6 mm^3^, with an average reduction of −0.04 ± 0.2 mm^3^. Between the two groups, no significant differences of the investigated baseline characteristics were observed (Table 2).

The exploratory analysis of possible influencing factors on the follow-up ETDRS letter score revealed a positive correlation with the baseline ETDRS letter score (*p* < 0.001) (Table 3). Additionally, the follow-up CRT showed a positive correlation with baseline CRT (*p* < 0.003), the number of prior anti-VEGF agents (*p* < 0.034) and the number of prior corticosteroid injections (*p* < 0.019) (Table 4). No other statistically significant correlations with the follow-up ETDRS letter score or follow-up CRT were observed.

No adverse events were recorded following the IVF injections.

## 4. Discussion

With our study, we showed immediate and significant improvements of structural measures, as reductions of CRT and CRV were observed, while an improvement of the visual acuity was not noted in the follow-up exam, following a therapy switch to Faricimab. In our subgroup analysis, we could not observe statistically significant differences between the defined Faricimab responders and reduced-responders. However, in a descriptive comparison, the mean values of the reduced-responder group showed a worse course of prior treatment history, including a longer period of time since the first anti-VEGF injection, a higher baseline number of intravitreal injections and previously applied anti-VEGF agents, a higher number of prior intravitreal corticosteroid and triamcinolone injections as well as a higher count of prior pars-plana vitrectomies and membrane peelings. The exploratory data analysis revealed a positive statistically significant influence of the baseline ETDRS letter score on the final visual acuity as well as, although not statistically significant, a noticeably negative correlation of the status of the diabetic retinopathy at baseline. The follow-up CRT was positively and statistically significant influenced by the baseline CRT, the number of prior anti-VEGF-agents as well as the number of prior intravitreal corticosteroid injections.

In contrast, the respective study cohorts show that some of the baseline patient characteristics are consistent between our investigation and the YOSEMITE and RHINE trial. In our study, the mean age (65 years), the percentage of patients affected by type-2 diabetes (100%), the mean HbA1c (7.5%) as well as mean baseline ETDRS score (59.4 letters) were similar to the patient cohort of the YOSEMITE and RHINE trial (age: 62.8 and 61.6 years; percentage of type-2 diabetes: 96% and 94%; HbA1c: 7.6% and 7.7%; baseline ETDRS score: 61.9 and 62.5 letters) [26]. On the other hand, there are some clear differences between our patient cohort, with regard to the percentage of females (18.7%), the number of treatment naïve patients (0%), the percentage of severe NPDR or PDR (80%) and the baseline CRT (414.4 µm) compared to the YOSEMITE and RHINE trial (percentage of females: 37% and 38%; the number of treatment naïve patients: 78% and 80%; percentage of severe NPDR or PDR 39% and 43%; baseline CRT: 485.8 and 471.3 µm) [26]. While the first intravitreal anti-VEGF injection before the switch to IVF in our study cohort was, on average, 50.4 months ago, in the YOSEMITE and RHINE trial, the diagnosis of DME was, on average, only 17.6 and 20.7 months earlier [26]. 

Contrary to our analysis, the YOSEMITE and RHINE trial showed significant improvements in visual function as early as 4 weeks following IVF [26]. In our analysis, an average improvement of 2 ETDRS letters was observed 3 months after switching to Faricimab, whereas in the YOSEMITE and RHINE trial, the gain of visual function was 9 and 8 letters after 3 months, respectively [26]. This discrepancy can be explained by the above-mentioned differences. It has been shown that factors such as prolonged DME, an extended period of anti-VEGF therapy and a worse status of DR at baseline exhibit a diminished potential of functional improvement, which is mainly attributed to structural alterations of the retina over time due to recurrent and chronic macula oedema [27,28,29].

The exploratory data analysis revealed that a higher ETDRS score at baseline was associated with a higher ETDRS score at follow-up. However, even though there were no functional improvements over all patients, the subgroup analysis revealed distinct differences between the responder group (Baseline ETDRS score: 53 letters; change of EDTRS score at follow-up: +10 letters), compared to the reduced-responder group (Baseline ETDRS score: 62; change of EDTRS score at follow-up: −1 letter). These observations are often described as the so-called “ceiling effect”, whereby patients that are treated with anti-VEGF injections over time are limited in the maximum visual gain possible [19,30]. Therefore, patients with a lower visual acuity at baseline can benefit from the treatment, whereas a higher visual function at baseline has less room for improvement before reaching the “ceiling”. Even though a correlation of the baseline ETDRS score and the change of ETDRS letters at follow-up was not statistically significant (*p* = 0.077), we can see a distinct correlation (see Supplementary, Figure S1).

Another retrospective analysis of patients affected by DME by Kusuhara et al. included both previously treated (33%) and treatment-naïve (67%) eyes and also observed no functional improvements, following 4 months of IVF [19]. In contrast, a retrospective study by Rush et al., which also only included previously treated eyes, demonstrated a significant improvement in visual acuity as a result of IVF, 4 months after the therapy switch [21]. These varying results have already been discussed by Kusuhara et al. and can again be attributed to the “ceiling effect” [19]. The baseline visual function of the study cohort of Rush et al. was thereby notably poorer (Baseline BCVA in LogMAR: 0.6; converted to the ETDRS letter score after Beck et al. [24]: 55 letters) compared to our cohort (Baseline ETDRS letter score: 59 letters) [21]. The visual acuity at the follow-up exam of Rush et al. (Follow-up BCVA in LogMAR: 0.5; converted to the ETDRS letter score after Beck et al. [24]: 60 letters), compared to our study (follow-up ETDRS letter score: 61 letters), showed similar results, following the therapy switch to IVF [21].

Inferior improvements of the visual function in comparison to the YOSEMITE and RHINE trial have also been described in further DME Faricimab real-world studies. In the TAHOE trial, in comparison to the YOSEMITE and RHINE trial, a higher proportion of pre-treated patients (44.7%) have been included while a functional improvement of only 4 letters was observed 3 months after the therapy switch [22]. In the FARETINA-DME study, 87.5% of the included patients were previously treated with anti-VEGF injections, whereas only a stable visual acuity was described at a 2-month follow-up examination [22,31].

Regarding anatomical changes, a significant reduction of the CRT was observed in our study, following the switch to Faricimab. Three months after the therapy switch, the YOSEMITE and RHINE study cohort showed a CRT < 325 µm in around 67% and 65%, whereas in our study, the CRT < 325 µm was achieved by 56% of all patients in a similar period [26]. Considering the previously mentioned differences in patient characteristics, particularly among the missing of treatment-naïve patients in our analysis compared to the YOSEMITE and RHINE study, it is important to note that lower anatomical improvements are to be expected [29,32]. Similar results of the CRT following IVF have also been described by Kusuhara et al. (329.8 µm) as well as Rush et al. (340.3 µm) 4 months after the therapy switch, compared to our analysis (353 µm), 3 months after the therapy switch [19,21]. 

In the exploratory data analysis, we observed that a larger baseline CRT, a higher number of previous anti-VEGF agents and prior intravitreal corticosteroid injections negatively influenced the post-IVF CRT. As described in the literature, these factors collectively suggest a history of prolonged duration of therapy with consistently poor anatomical outcomes [33,34]. 

The anatomical improvements we found may be explained by the novel dual inhibition mechanism of Faricimab [35,36]. Former anti-VEGF agents, such as Aflibercept, Bevacizumab, Ranibizumab and Brolucizumab, primarily inhibit the VEGF-A. Through the dual inhibition of VEGF-A and Ang-2, multiple pathophysiological pathways are tackled which proved to be effective in our study.

The limitations of our analysis include the small number of cases as well as the single center retrospective approach. Furthermore, the visual acuity was measured as decimal BCVA and, for a better comparison, converted to the ETDRS letter score, which is less precise than using ETDRS vision charts for the collection of data. Lastly, we only analyzed the short-term outcomes following the initial IVF series.

## 5. Conclusions

The switch to IVF resulted in a noticeable reduction of the CRT and CRV, but visual acuity did not show a statistically significant functional improvement across all patients. The subgroup analysis revealed that particularly patients with a worse baseline visual acuity can improve following IVF. A predictor for a good visual acuity following IVF was a good baseline visual function. A high initial CRT, a higher number of previous applied anti-VEGF agents, as well as a greater number of prior corticosteroid injections, negatively influenced the CRT following IVF. Even though a functional improvement was not observed, Faricimab proved to be safe and demonstrated stable visual acuity as well as effective anatomical improvements in the short term. Long-term data are necessary to evaluate especially further changes of the visual function.

## Figures and Tables

**Table 1:** Baseline patient characteristics.

Characteristics		*n* (%)
Number of eyes/Number of patients		25/16
Sex		
	male	13 (81.3)
	female	3 (18.7)
Age in years (mean ± SD)		65.0 ± 8.0
Laterality		
	right	11 (44.0)
	left	14 (56.0)
HbA1c in % (mean ± SD)		7.5 ± 1.2
Number of patients affected by type-2 diabetes		16 (100%)
Status of diabetic retinopathy		
	NPDR, mild/moderate/severe	0 (0)/5 (20.0)/15 (60.0)
	PDR	5 (20.0)
Years since first anti-VEGF injection (mean ± SD)		4.6 ± 3.1
Number of prior anti-VEGF agents (mean ± SD)		1.8 ± 0.8
Total number of prior anti-VEGF injections (mean ± SD)		24.6 ± 16.6
Total number of prior intravitreal dexamethasone injections (mean ± SD)		1.8 ± 2.8
Intravitreal therapy in prior year before Faricimab switch		
	Eyes treated with anti-VEGF therapy/dexamethason	19 (76.0)/6 (24.0)
	Number of anti-VEGF injections (mean ± SD)	6.2 ± 1.8
	Last anti-VEGF agent, Aflibercept/Ranibizumab	14 (56.0)/5 (20.0)
	Number of dexamethason injections (mean ± SD)	2.2 ± 0.4
Number of Faricimab injections in initial series (mean ± SD)		2.92 ± 0.6
Months since first Faricimab injection at follow-up		3.26 ± 0.8
Previous intravitreal triamcinolon therapy		4 (16.0)
Previous focal laser therapy		4 (16.0)
Lens status		
	phakia	9 (36.0)
	pseudophakia	16 (64.0)
Status after vitrectomy		6 (24.0)
Complications of vitreomacular traction		
	Epiretinal membrane	8 (32.0)
	Status after previous membrane peeling	5 (20.0)
Concomitant hypertension		12 (75)
Status after acute cardiovascular event		4 (25)

Note: Previous anti-VEGF agents include intravitreal injection of aflibercept, bevacizumab and ranibizumab; status after acute cardiovascular event is defined as at least one previous event of NSTEMI, STEMI or stroke. Abbreviations: SD, standard deviation; NPDR, non-proliferative diabetic retinopathy; PDR, proliferative diabetic retinopathy.

**Table 2:** Univariate analysis of treatment response groups.

		Responder (*n* = 7)	Reduced-Responder (*n* = 18)	*p*-Value
Sex (male/female)		6/1	13/5	0.478
Age in years (mean ± SD)		67.2 ± 6.7	64.8 ± 8.1	0.488
Laterality, right/left		2/5	9/9	0.332
HbA1c in % (mean ± SD)		7.3 ± 0.7	7.7 ± 1.5	0.528
Status of diabetic retinopathy				
	mild NPDR/moderat NPDR/severe NPDR/PRD	0/1/4/2	0/4/11/3	0.490
Baseline ETDRS letters (mean ± SD)		52.9 ± 18.5	61.9 ± 10.5	0.131
	Changes in ETDRS letter (mean ± SD)	10 ± 7.6	−1.1 ± 4.7	
Baseline CRT (mean ± SD)		464.6 ± 113.1	394.4 ± 128.7	0.223
	Changes in CRT (mean ± SD)	−194.4 ± 99.3	−9.3 ± 49.4	
Baseline 3 mm CRV (mean ± SD)		3.1 ± 0.5	2.7 ± 0.6	0.180
	Changes of 3 mm CRV (mean ± SD)	−0.86 ± 0.4	−0.04 ± 0.2	
Years since first anti-VEGF injection (mean ± SD)		4.2 ± 2.4	4.8 ± 3.3	0.690
Number of prior anti-VEGF injections (mean ± SD)		23.4 ± 15.9	25 ± 17.4	0.837
Number of prior anti-VEGF agents (mean ± SD)		1.7 ± 0.8	1.9 ± 0.8	0.634
Number of Faricimab injections in initial series (mean ± SD)		3.0 ± 0.6	2.9 ± 0.7	0.706
Number of prior dexamethasone injections (mean ± SD)		0.7 ± 1.5	2.2 ± 3.1	0.120
Prior triamcinolon injection, yes/no		0/7	4/14	0.174
Lens status, phakia/pseudophakia		2/5	7/11	0.629
Status after vitrectomy, yes/no		1/6	5/13	0.478
Prior membrane peeling, yes/no		1/6	4/14	0.656
Previous focal laser therapy, yes/no		0/7	4/14	0.174
Previous panretinal photocoagulation, yes/no		4/3	9/9	0.748

Note: Responder group defined as gain of ≥ 5 ETDRS letters as well as reduction of the CRT of ≥30 µm. Abbreviations: ETDRS, Early Treatment of Diabetic Retinopathy Study; SD, standard deviation; NPDR, non-proliferative diabetic retinopathy; PDR, proliferative diabetic retinopathy.

**Table 3:** Explorative analysis on the follow-up ETDRS letter score following IVF.

	Correlation Coefficient	*p*-Value
Age	−0.030	0.885
Status of diabetic retinopathy	−0.378	0.063
Baseline ETDRS letters	0.764	<0.001 *
Years since first anti-VEGF injection	−0.238	0.275
Number of prior anti-VEGF injections	−0.155	0.459
Number of prior anti-VEGF agents	0.166	0.427
Number of previous corticosteroid injections	−0.195	0.351

Abbreviations: ETDRS, Early Treatment of Diabetic Retinopathy Study; VEGF, vascular endothelial growth factor; IVF, intravitreal Faricimab; * Statistically significant.

**Table 4:** Explorative analysis on the follow-up CRT following IVF.

	Correlation Coefficient	*p*-Value
Age	0.155	0.459
Status of diabetic retinopathy	−0.060	0.677
Baseline CRT	0.571	0.003 *
Years since first anti-VEGF injection	0.137	0.532
Number of prior anti-VEGF injections	0.170	0.416
Number of prior anti-VEGF agents	0.425	0.034 *
Number of previous corticosteroid injections	0.467	0.019 *

Abbreviations: CRT, central retinal thickness; VEGF: vascular endothelial growth factor; IVF, intravitreal Faricimab; * Statistically significant.

## Data Availability

Due to privacy restrictions, the data contained within this manuscript is not publicly accessible, except for the data that has been published.

## Supplementary Materials

The following supporting information can be downloaded at: https://www.mdpi.com/article/10.3390/jcm13154508/s1, Table S1. Treatment outcomes following IVF. Figure S1. Correlation of baseline ETDRS letter score and change of ETDRS letter score at follow-up. Correlation coefficient (Pearson): −0.360; *p* = 0.077.
