# Supplementary material for: Short-Term Clinical Outcomes of Patients with Diabetic Macular Edema Following a Therapy Switch to Faricimab
Source: J Clin Med. 2024 Aug 1;13(15):4508. doi: 10.3390/jcm13154508 (PMC11312860; doi:10.3390/jcm13154508)
Supplement: Supplementary file 1 [file jcm-13-04508-s001.zip › jcm-3115869-supplementary.pdf]

Journal of
*Clinical Medicine*

MDPI

## Supplementary

**Table S1.** Treatment outcomes following IVF (Supplementary Table for Figure 1).

| | **Mean ± SD.** | | | |
|---|---|---|---|---|
| | Pre-Faricimab | Post-Faricimab | Difference ± SD | *p*-Value |
| ETDRS letters | 59.4 ± 13.4 | 61.4 ± 12.8 | 2.0 ± 7.5 | 0.26 |
| CRT (µm) | 414.4 ± 126.3 | 353.3 ± 131.1 | −61.2 ± 106.7 | 0.011 * |
| 3mm CRV ($mm^3$) | 2.8 ± 0.5 | 2.6 ± 0.6 | −0,27 ± 0.47 | 0.012 * |

**Note**: Comparison between the pre- and post-Faricimab therapy switch of the CRT, CRV and 3mm CRV. **Abbreviations:** ETDRS**,** Early Treatment of Diabetic Retinopathy Study; SD, Standard deviation; CRT, central retinal thickness; CRV, central retinal volume; IVF, intravitreal Faricimab. * Statistically significant.

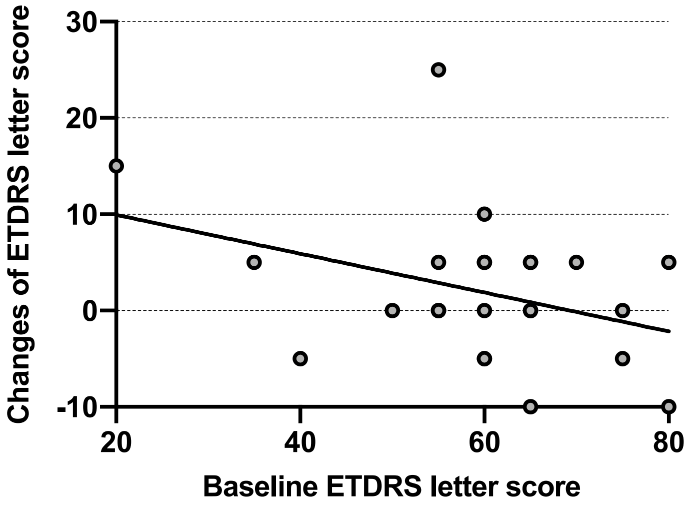


**Figure S1.** Correlation of baseline ETDRS letter score and change of ETDRS letter score at follow-up. Correlation coefficient (Pearson): −0.360; $p = 0.077$. **Abbreviations:** ETDRS**,** Early Treatment of Diabetic Retinopathy Study.
